# Percutaneous angio-embolization of a post laparoscopy complex utero-adenexal vascular malformation

**DOI:** 10.4103/0972-9941.41950

**Published:** 2008

**Authors:** Ashish Verma, Suyash Mohan, Tripti Chandra, Manoj K Kathuria, Sanjay Saran Baijal

**Affiliations:** Department of Radiodiagnosis, Sanjay Gandhi Postgraduate Institute of Medical Sciences, Raebareily Road, Lucknow - 226 014, India

**Keywords:** Embolisation in pelvic vascular malformations, laparoscopic complications, uterine AVM

## Abstract

Vascular abnormalities are uncommon causes of uterine bleeding. Laparoscopic surgeries, however, require expertise and improper techniques can lead to major vascular complications. We report an unusual case of utero-adenexal arterio- venous fistula with arterio - venous malformation due to pelvic trauma caused during laparoscopic sterilisation procedure, which was treated by percutaneous embolisation technique. To the best of our knowledge, this is the first documentation of such a complex vascular injury caused by laparoscopic sterilisation and its endovascular management.

## INTRODUCTION

Vascular abnormalities are rare but potentially life-threatening causes of uterine bleeding in women of reproductive age,[1] especially in women with past history of infection, curettage, therapeutic abortion, use of intra-uterine contraceptive devices, endometrial carcinoma, gestational trophoblastic disease and pelvic surgeries.[2] Since the advent of minimally invasive surgery the incidence of such complications has significantly reduced. Early diagnosis and treatment in such cases is crucial because of potentially fatal outcome due to intractable bleeding. We report an unusual case of utero-adenexal arterio-venous fistula (AVF) with arterio-venous malformation (AVM) due to pelvic trauma caused during laparoscopic sterilization procedure, which was treated by percutaneous embolisation technique.

## CASE REPORT

**Figure 1:**
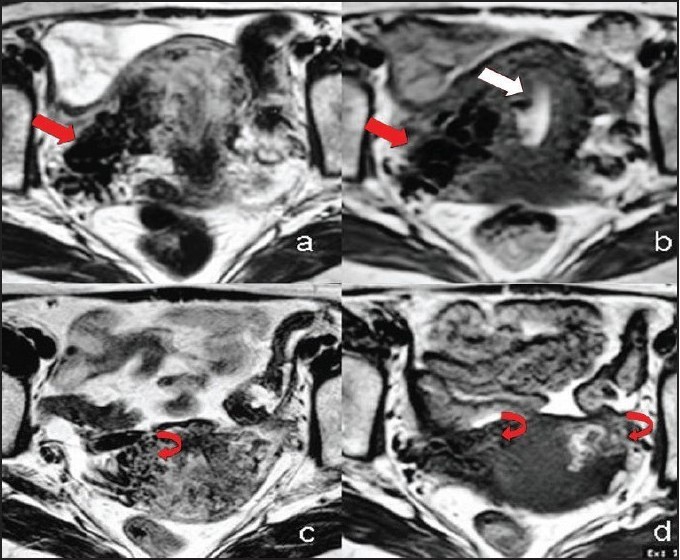
Pre-embolisation Axial T2W (a) and T1W (b) MRI scans at the level of uterine body and cervix show presence of multiple tortuous flow voids suggesting abnormal vessels in the myometrium and adenexa (solid arrows). More number of abnormal vessels is seen on the right side. Also blood (hollow arrow) is seen in the endometrial cavity (b). Comparable post-embolisation Axial T2W (c) and T1W (d) MRI scans show thrombosed vessels (curved arrows)

**Figure 2:**
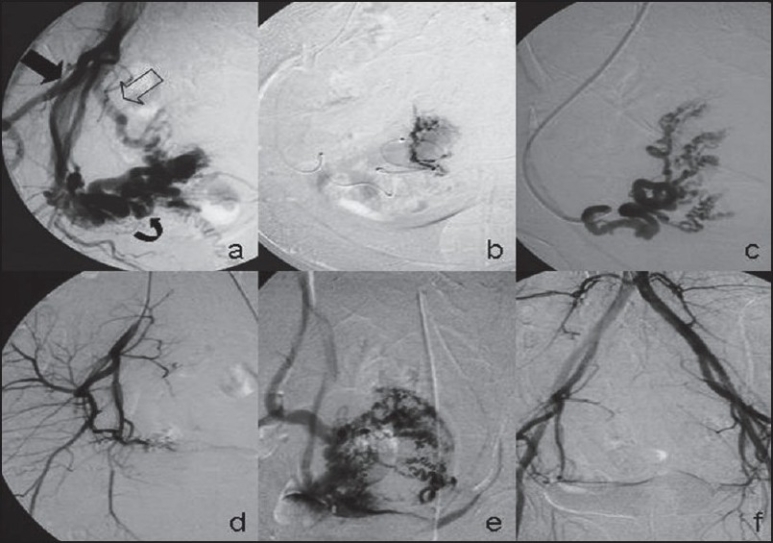
Digital substraction angiography series shows (a) an extra uterine abnormal vascular tangle in the right adenexa (curved arrow) with arterio-venous fistula showing early filling of pelvic vein (hollow arrow) on right internal iliac artery (solid arrow) injection. (b) Selective cannulation of the fistula with a microcatheter- wire combination was done followed by injection of histoacryl glue. (c) Contrast injection in the distal uterine artery shows right sided myometrial arterio-venous malformation. (d) Check angiogram following embolisation of the myometrial arterio-venous malformation shows disappearance of the lesion. (e) Contralateral uterine artery injection shows feeders to the arterio-venous malformation from the left side as well. This was followed by PVA particle embolisation from left side also. (f) Final check fl ush aortogram shows no filling of the malformation or fistula.

A 35-year-old female presented with profuse, irregular, intermittent vaginal bleeding for the past six months. One month prior to onset of the symptoms she underwent a laparoscopic tubal ligation at a peripheral centre. Her past obstetric history was non-contributory. General, systemic and gynaecological examinations were unremarkable except for the presence of significant pallor. Abdominal and transvaginal sonography showed presence of multiple vascular channels in the parametrium and myometrium with a bulky uterus and fluid in the endometrial cavity. Color Doppler imaging, suggested presence of arterio-venous shunting. Pelvic magnetic resonance imaging (MRI) [Figure 1 a, b] revealed presence of multiple flow voids in the myometrium and parametrium, more on the right, with features suggesting blood within the cavity. A diagnosis of complex utero-adnexal arterio-venous malformation with suspected fistula was considered and pre-hysterectomy angio-embolisation was planned. Bilateral common, internal and external iliac artery injections were acquired by left femoral puncture [Figure 2 a-f]. Abnormal vascularity supplied by uterine arteries was noted. Selective injection of right uterine artery showed high flow AVF with early drainage into pelvic veins and inferior vena cava, with hypertrophied radial and spiral arteries. More distally abnormal uterine parenchymal blush with tuft of vessels was noted, with cross filling from opposite uterine artery injection as well. To close the high flow AVF first, histo-acryl glue [in a ratio of 1:1 with contrast media] was injected through a cobra catheter, which was placed in the right uterine artery, very close to the origin of the high-flow AVF. Glue was injected after confirming free flow and ensuring that no major branch was visualised on the angiogram. Deep catheterisation obviated the need for using a micro-catheter. A microcatheter-wire system was subsequently advanced in the major feeders to the myometrial low flow fistulae using cobra catheter as the guiding catheter. Polyvinyl alcohol [P.V.A] particles (average size 500-750µ) (Cook Bloomington Inc, USA) were injected from both uterine arteries to achieve occlusion of this malformation. The procedure was terminated when no further filling of malformation was seen from either side. Patient was discharged on the second post-procedure day. The patient opted against hysterectomy as she became asymptomatic. Follow-up MRI showed occlusion of abnormal vascularity [Figure 1 c, d]. Similar symptoms, however, recurred after one and a half years for which a second sitting of angio-embolisation was done. Hysterectomy was done on the fourth day of embolisation this time. Gross operative specimen and cut section showed few abnormal vascular spaces in the myometrium. The patient was asymptomatic till last follow-up.

## DISCUSSION

Durreil and Loubat first described uterine AVM in 1926[3] and classified them into congenital and acquired types.[4] The acquired uterine AVM occurs due to previous uterine surgery, dilatation and curettage, previous pregnancy-related trauma, gestational trophoblastic diseases, exposure to diethyl-stilbesterol, endometriosis, fibromyoma and cervical or endometrial cancers. Acquired AVMs are actually multiple small arterio-venous fistulae between intramural arterial branches and the myometrial venous plexus.[5] They can have feeder from single or bilateral uterine arteries, but no supply from extra-uterine arteries exists. Congenital AVM, unlike acquired AVMs, tend to have multiple feeding arteries and draining veins and an intervening nidus with invasion into the surrounding structures like muscles or adjacent viscera. Therefore, it is much easier to treat acquired AVM than a congenital AVM.[6] AVF are usually acquired lesions and occur as a consequence of a previous trauma.[7] In the present case, the laparoscopy was done by the old technique, and a suspicion of vascular injury during insertion of the trochar was raised. The most common presentation of uterine vascular malformations is recurrent vaginal bleeding which is resistant to treatment. Pattern of bleeding is usually intermittent and torrential, occasionally significant enough to require blood transfusions. Our patient had a typical symptomatology but the diagnosis was delayed due to low index of suspicion. Such bleeding presumably occurs when sub-endometrial vessels gets exposed to cavity during menstruation or are traumatized iatrogenically.[4]

Ultrasound with Color Doppler remains the primary imaging modality, which shows many hypoechoic or anechoic spaces, with evidence of flow on colour Doppler. Angiography is the gold standard for diagnosing these lesions and is essential to landmark the pattern of blood supply to the lesion pre-operatively. In addition, percutaneous endovascular embolisation preceding surgery can help contain the per-operative blood loss. Angiographic features consist of a complex tangle of vessels supplied by enlarged feeders from the uterine arteries. Early venous drainage indicated an associated AVF[6] as was seen in our patient.

Although laparoscopy is a relatively safe procedure occasionally serious complications such as major vascular injury (0.1 per 1000) arise.[8] Such cases can be managed conservatively, but most surgeons prefer a more aggressive approach of early hysterectomy. Presently percutaneous endovascular embolisation is the therapy of choice for AVMs and AVFs, with many advantages including high success, lower complications, avoidance of surgical risks, retained reproductive capacity and restoration of normal menstruation.[8] Our patient was treated by intra-arterial embolisation of both uterine arteries with glue and PVA particles, with prompt cessation of bleeding after the first sitting. Recurrence of the pathology, probably due to large initial size, eventually led to hysterectomy. We submit that the recurrence in the present case was due to large size of the lesion and not the inadequacy of procedure. Unwarranted delay in hysterectomy, like in this case may lead to opening up of potential feeders and hence, recurrence of symptoms.

Endovascular embolotherapy may serve as a viable option for management of vascular complications secondary to surgical trauma. The present report highlights a rare vascular complication due to a minimally invasive technique which was managed by another minimally invasive endovascular approach.
